# Antinociceptive effects of vitexin in a mouse model of postoperative pain

**DOI:** 10.1038/srep19266

**Published:** 2016-01-14

**Authors:** Qing Zhu, Li-Na Mao, Cheng-Peng Liu, Yue-Hua Sun, Bo Jiang, Wei Zhang, Jun-Xu Li

**Affiliations:** 1 School of Pharmacy, Nantong University, Nantong, 226001, Jiangsu Province, China

## Abstract

Vitexin, a C-glycosylated flavone present in several medicinal herbs, has showed various pharmacological activities including antinociception. The present study investigated the antinociceptive effects of vitexin in a mouse model of postoperative pain. This model was prepared by making a surgical incision on the right hindpaw and von Frey filament test was used to assess mechanical hyperalgesia. Isobolographical analysis method was used to examine the interaction between vitexin and acetaminophen. A reliable mechanical hyperalgesia was observed at 2 h post-surgery and lasted for 4 days. Acute vitexin administration (3–10 mg/kg, i.p.) dose-dependently relieved this hyperalgesia, which was also observed from 1 to 3 days post-surgery during repeated daily treatment. However, repeated vitexin administration prior to surgery had no preventive value. The 10 mg/kg vitexin-induced antinociception was blocked by the opioid receptor antagonist naltrexone or the GABA_A_ receptor antagonist bicuculline. The doses of vitexin used did not significantly suppress the locomotor activity. In addition, the combination of vitexin and acetaminophen produced an infra-additive effect in postoperative pain. Together, though vitexin-acetaminophen combination may not be useful for treating postoperative pain, vitexin exerts behaviorally-specific antinociception against postoperative pain mediated through opioid receptors and GABA_A_ receptors, suggesting that vitexin may be useful for the control of postoperative pain.

Postoperative pain remains a major clinical problem after many surgeries. The mismanagement of postoperative pain brings about a variety of negative consequences, such as chronic postsurgical pain and delayed rehabilitation^1^. Despite the advances in the understanding of the pathophysiology mechanisms of postoperative pain and the use of preventative strategies and analgesic agents, including opioids, nonsteroidal anti-inflammatory drugs (NSAIDs), adjuvant drugs, and topical anesthesia, postoperative pain continues to be under-treated and most of the surgical patients still suffer from moderate to severe postoperative pain^2^,^3^. The analgesic agents widely used in clinical treatment usually have limited effectiveness and safety. For example, NSAIDs are associated with serious gastrointestinal lesions or renal and liver failure. The opioids remain the most effective analgesics for pain management, but their use is controversial and restricted due to their perceived side effects and fears of addiction and tolerance. Therefore, further studies are required to develop novel and effective alternative medicine for pain treatment and preventative strategies.

Vitexin, a C-glycosylated flavone (5, 7, 4-trihydroxyflavone-8-glucoside), is a major bioactive ingredient of the traditional Chinese herb *Crataegus pinnatifida* (hawthorn)^4^, which has also been identified in several other medicinal herbs^5^. The anti-inflammatory and anti-hypertensive effects of vitexin were first described in 1980s^6^. Recently, vitexin has gained an increasing attention because more pharmacological potentials of vitexin have been found in past decades. Accumulating evidence has demonstrated that vitexin possesses antioxidant^7^,^8^,^9^, antitumor^10^,^11^,^12^, antiviral^13^,^14^, anti-spasmodic^15^, antithyroid^16^,^17^, and cardioprotective properties^18^,^19^,^20^. In addition, vitexin has also been found to have beneficial effects on neurological dysfunction. For example, vitexin has anti-depressant-like effects in mice^21^, ameliorates scopolamine-induced memory impairments in rats^22^, and exerts neuroprotective effects on several brain injury models^23^,^24^,^25^. Limited evidence from recent studies suggests that vitexin also has pain-relieving abilities. Acute administration of vitexin attenuates the writhing response of mice induced by chemical stimuli such as acetic acid^26^,^27^,^28^ and phenyl-p-benzoquinone (PBQ)^28^. Vitexin also exerts modest antinociceptive activities against noxious thermal stimuli in hot-plate test and mechanical stimuli in tail-clip test. Interestingly, the antinociceptive effects of vitexin are able to be reversed by delta, mu, and kappa opioid receptor antagonists respectively, suggesting that the antinociceptive effects are mediated by opioid-related mechanisms^27^. In addition, vitexin inhibits the pain-like behaviors and reduces the mechanical and thermal hyperalgesia in a variety of inflammatory pain mice models. The antinociceptive effects of vitexin against inflammatory pain may be partially mediated through targeting transient receptor potential vanilloid 1 (TRPV1) channel and oxidative stress and modulating cytokine production^28^. However, whether vitexin could relieve postoperative pain and its underlying mechanisms remain unclear. In order to provide further evidence for the clinical application of vitexin as an analgesic agent, this study investigated the antinociceptive effects of vitexin in a mouse model of postoperative pain and explored its potential pharmacological mechanisms. In addition, we also examined the preventive effect of vitexin against postsurgical pain. It is well known that acetaminophen (paracetamol) is often used alone or in combination with traditional analgesics to treat postoperative pain; thus this study also examined the interaction of vitexin combined with acetaminophen on postoperative pain.

## Results

### Effects of vitexin on incision-induced mechanical hyperalgesia in mice

**Figure 1:**
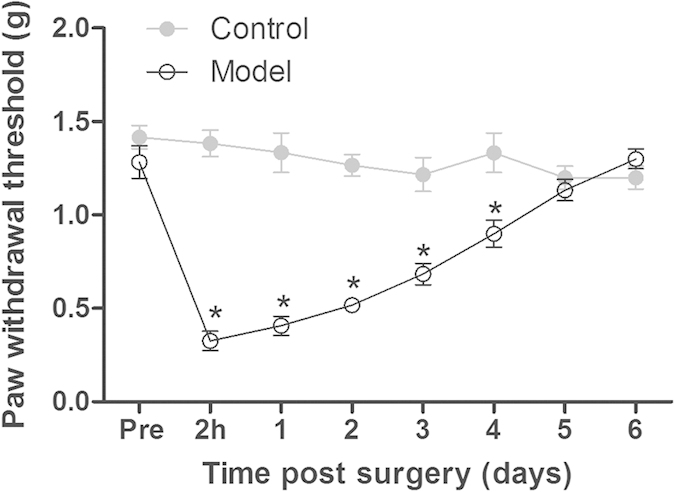
The time course of incisional surgery induced mechanical hyperalgesia in mice. Control: control group that did not receive surgery. Model: model group that received surgery. Data were expressed as mean ± SEM (n = 12 per group), assessed by two-way ANOVA with repeated measures followed by Bonferroni post hoc analysis. *P < 0.001 compared to the corresponding data of control group. There was no differences in baseline mechanical paw withdrawal threshold (PWT) between control and model group pre-surgery (Pre). The incisional surgery induced mechanical hyperalgesia was observed at 2 h post-surgery and lasted for at least 4 days.

Before the incisional surgery, there were no significant differences in baseline mechanical paw withdrawal threshold (PWT) between control group and model group (1.42 ± 0.06 g vs 1.28 ± 0.09 g, P > 0.05). Two hours after surgery, the PWT in model group was dramatically decreased, and then gradually recovered to the pre-surgery baseline level 5 days after surgery (Fig. 1). Two way ANOVA analysis showed significant main effects of surgery (F [1, 22] = 79.49, P < 0.0001), time (F [6, 132] = 14.62, P < 0.0001), and interaction between time and surgery (F [6, 132] = 26.09, P < 0.0001). Post hoc analyses revealed that the PWT was markedly lower in model group as compared to control group from 2 h to 4 days after surgery (P < 0.001). However, the PWT in control group remained at the baseline level under repeated measurement with von Frey filaments throughout the experimental period (gray filled circle, Fig. 1).

**Figure 2:**
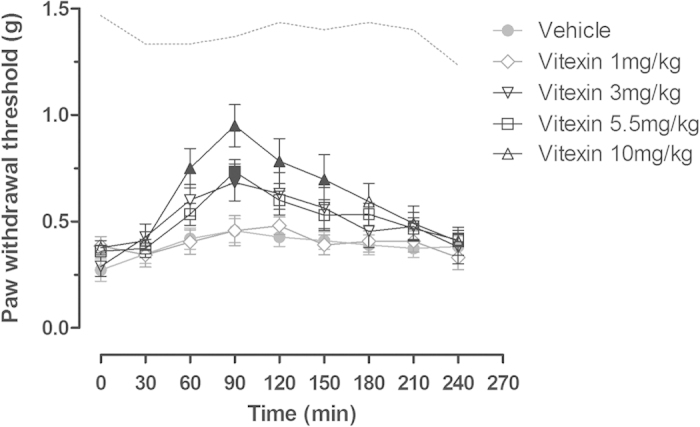
Effect of acute vitexin treatment on incision-induced mechanical hyperalgesia in mice. Data were expressed as mean ± SEM (n = 12 per group), assessed by two-way ANOVA with repeated measures followed by Bonferroni post hoc analysis. Filled black symbols indicated data significantly different from the corresponding vehicle group. Dashed line indicated the average PWT of the left hind paw from the vehicle group (P < 0.05).

At doses of 1–10 mg/kg, acute vitexin administration dose-dependently increased the PWT in the mice after incisional surgery (Fig. 2). In vehicle group that received incisional surgery and vehicle treatment, the PWT remained at a low level during repeated measurements within the 4-h test period (gray filled circle, Fig. 2). In contrast, the PWT gradually increased to the peak at 90 min and subsequently decreased to pre-treatment level at 4 h after the administration of different doses of vitexin. Two way ANOVA analysis revealed significant main effects of time and vitexin treatment (F [8, 440] = 28.54, P < 0.0001; F [4, 55] = 3.31, P < 0.05, respectively), and interaction between time and vitexin treatment (F [32, 440] = 2.66, P < 0.0001). *Post hoc* analyses indicated that the PWT was significantly increased at 90 min after 3 mg/kg and 5.5 mg/kg vitexin administration and between time 60 and 150 min after 10 mg/kg vitexin treatment (P < 0.05).

**Figure 3:**
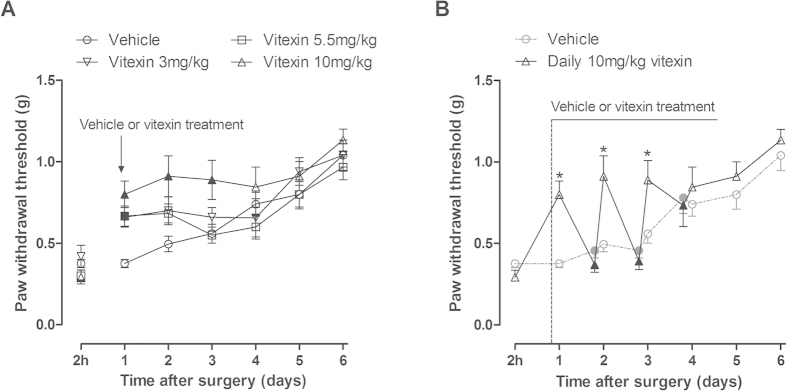
Effects of repeated vitexin treatment on incision-induced mechanical hyperalgesia in mice. (**A**) Daily treatment with vitexin (3–10 mg/kg) improved incision-induced mechanical hyperalgesia in mice in a dose-dependent manner. Data were expressed as mean ± SEM (n = 10–12 per group), assessed by two-way ANOVA with repeated measures followed by Bonferroni post hoc analysis. Filled black symbols indicated data significantly different from the corresponding vehicle group (P < 0.05). (B) Daily treatment with 10 mg/kg vitexin has no accumulative analgesic effect on incision-induced mechanical hyperalgesia in mice. *P < 0.05 compared to the corresponding data of vehicle group. Filled black symbols indicated the PWTs prior to drug administration at 2, 3, 4 day after surgery during the period that mice received daily 10 mg/kg vitexin treatment, which was not different from the corresponding vehicle group.

Since treatment of vitexin at a dose of 1 mg/kg had no anti-hyperalgesic effect, next we examined the effects of repeated vitexin administration at doses of 3–10 mg/kg against incisional pain in mice. Daily treatment with vitexin improved incision-induced mechanical hyperalgesia in mice in a dose-dependent manner (Fig. 3A). Two way ANOVA analysis revealed significant main effects of time and vitexin treatment (F [5, 185] = 19.87, P < 0.0001; F [3, 37] = 4.58, P < 0.01, respectively), but no significant interaction between time and vitexin treatment (F [15, 185] = 1.61, P > 0.05). *Post hoc* analyses indicated that daily 3 mg/kg or 5.5 mg/kg vitexin administration increased the PWT at 1 day post-surgery and daily 10 mg/kg vitexin treatment remarkably attenuated mechanical hyperalgesia from 1 to 3 days post-surgery (P < 0.05). In addition, when we measured the PWT prior to drug administration from 2 to 4 days post-surgery during the period that mice received daily 10 mg/kg vitexin treatment, the PWT was not significantly different from the vehicle-treated mice that never received drug treatment (Fig. 3B), suggesting that daily 10 mg/kg vitexin treatment has no accumulative effect and does not carry over the effect to the next day.

**Figure 4:**
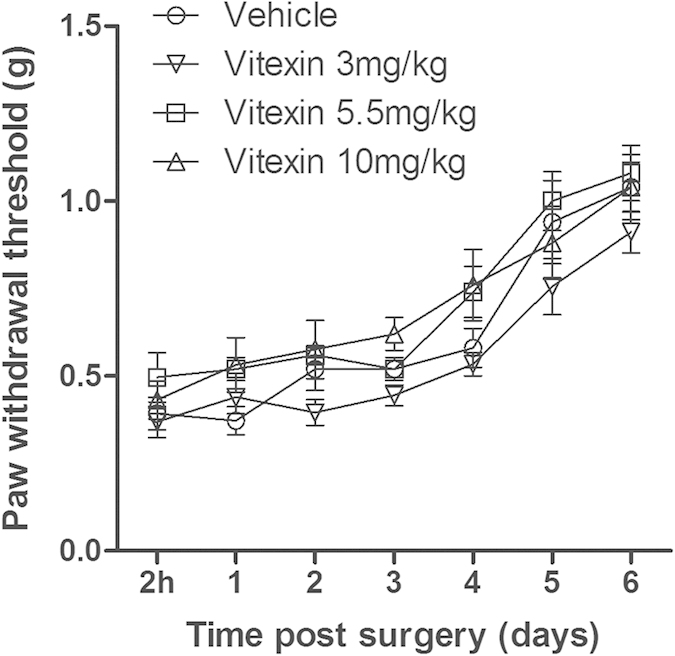
Effects of pre-surgery repeated vitexin treatment on the incision-induced mechanical hyperalgesia in mice. Data were expressed as mean ± SEM (n = 10 per group), assessed by two-way ANOVA with repeated measures followed by Bonferroni post hoc analysis. Repeated vitexin treatment for 6 days prior to incisional surgery did not alter the mechanical hyperalgesia in mice after surgery.

In an effort to examine whether repeated pre-treatment with vitexin has preventive effect against postoperative pain, we treated mice with daily vitexin for 6 days prior to incisional surgery. Such a treatment did not alter the PWT and produced no preventive effect against postoperative pain (Fig. 4). Two way ANOVA analysis did not show any statistical significance among mice with or without vitexin treatment.

### Studies using pharmacological antagonists

**Figure 5:**
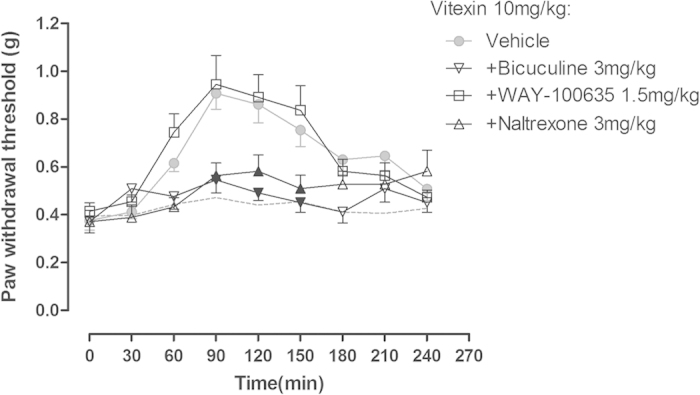
Effects of pretreatment with different receptor antagonists on the anti-hyperalgesic effects of vitexin (10 mg/kg) in mice receiving incisional surgery. Data were expressed as mean ± SEM (n = 10–12 per group), assessed by two-way ANOVA with repeated measures followed by Bonferroni post hoc analysis. Filled black symbols indicated data significantly different from the corresponding vehicle group (P < 0.05).Both the opioid receptor antagonist naltrexone and the GABA_A_ receptor antagonist bicuculline but not the 5-HT_1A_ receptor antagonist WAY100635 completely blocked the anti-hyperalgesic effects of 10 mg/kg vitexin.

In order to examine the potential pharmacological mechanisms of antinociception produced by vitexin against postoperative pain, we investigated the effects of pretreatment with different receptor antagonists on the antinociceptive effects of 10 mg/kg vitexin. As shown in Fig. 5, both 3 mg/kg naltrexone (an opioid receptor antagonist) and bicuculline (a GABA_A_ receptor antagonist) completely blocked the antinociceptive effects of 10 mg/kg vitexin in von Frey filament test. In contrast, 1.5 mg/kg WAY100635 (a serotonin 5-HT_1A_ receptor antagonist) did not alter the effects of vitexin. Two-way ANOVA showed significant main effects of the time (F [8, 336] = 21.90,  < 0.0001), antagonist treatment (F [3, 42] = 5.94, P < 0.01), and time × antagonist treatment interaction (F [24, 336] = 3.96, P < 0.0001). *Post-hoc* analyses indicated that naltrexone or bicuculline significantly prevented the antinociception of vitexin between 90–150 min after administration (P < 0.05).

### Effects of vitexin on the spontaneous locomotor activity in mice

**Figure 6:**
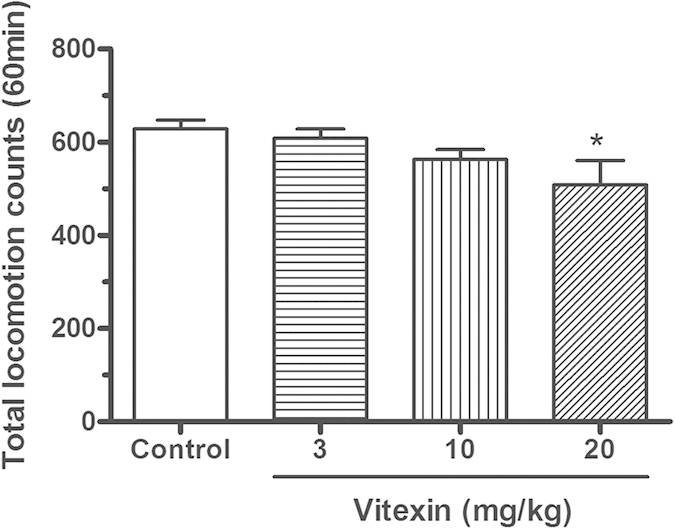
Effects of vitexin on the spontaneous locomotor activity in healthy mice. Data were expressed as mean ± SEM (n = 12 per group), assessed by one-way ANOVA followed by Student–Newman–Keuls post hoc analysis. *P < 0.05 as compared to control group that did not receive vitexin treatment. Acute vitexin treatment at dose not more than 10 mg/kg did not significantly suppress the general locomotor activity, but increasing the dose to 20 mg/kg reduced the locomotor activity in mice.

We measured the effects of different doses of vitexin on the spontaneous locomotor activity in mice to exclude possible nonspecific behavioral suppression of vitexin. It was found that acute vitexin treatment at doses of smaller than 10 mg/kg did not significantly alter the general locomotor activity; however, increasing the dose to 20 mg/kg produced significant hypolocomotion (one-way ANOVA: F [3, 44] = 3.44, P < 0.05). *Post hoc* analyses revealed that 20 mg/kg of vitexin significantly reduced the locomotor activity in mice (P < 0.05) (Fig. 6).

### Interactions between vitexin and acetaminophen

**Figure 7:**
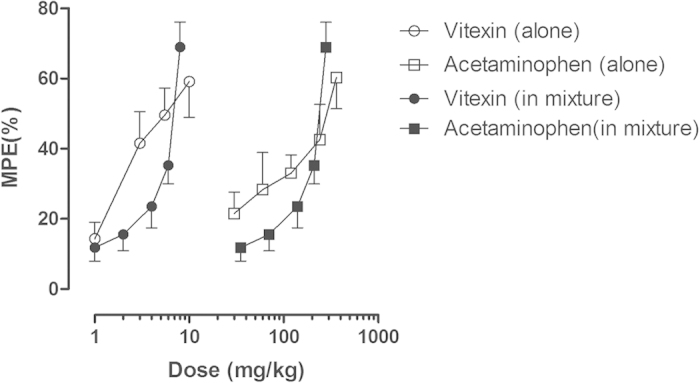
Anti-hyperalgesic effect of vitexin and acetaminophen alone or in the drug mixture (vitexin-acetaminophen combinations in a fixed proportion of 1:1) in mice receiving incisional surgery. Data were expressed as percentage of maximal possible effect (MPE) (mean ± SEM, n = 10–12 per group) and plotted as a function of drug dose; 100% MPE represented data from the pre-surgery baseline mechanical PWT.

**Figure 8:**
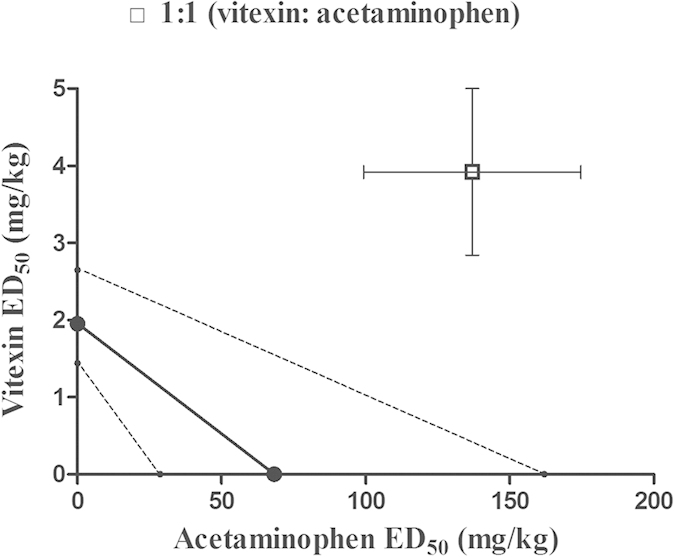
Isobologram showing the anti-hyperalgesic interaction of vitexin (ED_50_ = 1.95 mg/kg) and acetaminophen (ED_50_ = 68.27 mg/kg) in the mouse model of incisional pain. Abscissa scale: ED_50_ value of acetaminophen; Ordinate scale: ED_50_ value of vitexin (n = 10 per group). Horizontal and vertical bars indicate SEM. The oblique line between the x- and y-axes is the theoretical line of additivity. The dashed thin lines are the global 95% confidence boundaries, indicating the limits of the additive line. The point that represents ED_50_ values (±SEM) of the drug mixture fell far above the limits of the additive line, suggesting a significant infra-additive interaction.

We also examined the interaction between the antinociceptive effects of vitexin and acetaminophen on postoperative pain. Considering that 20 mg/kg vitexin produced hypolocomotive effects in mice and 500 mg/kg acetaminophen can induce hepatotoxicity in mice^29^, we limited the maximum dose of vitexin as 10 mg/kg and acetaminophen as 360 mg/kg. In our study, the doses of 1, 3, 5.5, 10 mg/kg of vitexin and 30, 60, 120, 240, 360 mg/ mg/kg of acetaminophen both produced dose-dependent anti-hyperalgesic effects (duration of action of the anti-hyperalgesic effects of acetaminophen were not shown), and the maximal effects of these individual doses were used to construct the dose-effect curves to calculate respective ED_50_ values of the two drugs. The anti-hyperalgesic effects of drugs were represented as MPE (%), and the ED_50_ (±SEM) values of vitexin or acetaminophen alone were determined from the MPE (%) of each drug dose respectively (Fig. 7). Vitexin produced a maximum of 59.1% MPE and acetaminophen produced a maximum of 60.3% MPE in the von Frey test. The ED_50_ (±SEM) value of vitexin was calculated as 1.95 ± 0.14 mg/kg (95% CIs: 1.44–2.65 mg/kg) and the ED_50_ (±SEM) value of acetaminophen was 68.27 ± 18.77 mg/kg (95% CIs: 28.77–162.00 mg/kg) using the nonlinear regression curve-fitting method. Then different doses of vitexin and acetaminophen were studied in combination based on the respective ED_50_ values (i.e., 0.5-, 1-, 2-, 3- and 4-folds of ED_50_ values). The maximal effects of the respective dose combinations were used to plot the dose-effect curve, and the respective ED_50_ values were calculated for vitexin and acetaminophen using the new combined dose-effect curve (Fig. 7). The ED_50_ value (±SEM) of vitexin was increased from 1.95 ± 0.14 mg/kg (vitexin alone) to 3.92 ± 1.08 mg/kg (vitexin-acetaminophen combination). The ED_50_ value (±SEM) of acetaminophen was increased from 68.27 ± 18.77 mg/kg (acetaminophen alone) to 137.0 ± 37.74 mg/kg (vitexin-acetaminophen combination). Isobolographical analysis indicated that the point that represents ED_50_ values (±SEM) of the drug mixture fell far above the limits of the line of additivity, suggesting that the interaction between vitexin and acetaminophen was infra-additive or antagonistic (Fig. 8).

## Discussion

In the present study, we for the first time found that both acute and repeated vitexin treatment had significant antinociceptive effects against postoperative pain. Although repeated administration before surgery seems to have no preventive effect on postsurgical pain, the positive findings on antinociceptive effects produced by acute or repeated vitexin treatment suggest that vitexin may be useful as a new alterative agent for the management of postoperative pain. Importantly, the antinociceptive effects of vitexin were able to be reversed by pretreatment with naltrexone or bicuculline, suggesting that both opioid receptors and GABA_A_ receptors contribute to the antinociception of vitexin on postoperative pain. Surprisingly, we found that the interaction between vitexin and acetaminophen was infra-additive, which indicated that it is not advisable to control postsurgical pain using vitexin in combination with acetaminophen.

Postoperative pain remains a clinical problem and limited preventive strategies or analgesic agents have been proven effective in preventing and relieving this type of pain. Novel alterative agents and strategies are highly attractive^1^,^2^,^3^. Despite limited evidence indicating that vitexin exerts antinociceptive activities against noxious stimuli in healthy animals^27^ and exhibits analgesic effects on an inflammatory pain model^28^, whether vitexin can manage postoperative pain remains unknown. Previous studies have developed a mouse model of incisional pain which has been recognized as a reliable and useful tool for drug discovery and the study of neurobiological mechanisms of postoperative pain^30^,^31^. Thus, in this study we applied a mouse model of incision-induced pain to investigate the antinociceptive effects of vitexin. It was found that the incision-induced mechanical hyperalgesia was observed at 2 h post-surgery and lasted for at least 4 days, which is consistent with previous reports^30^,^31^. Acute vitexin treatment markedly ameliorated mechanical hyperalgesia in this model of postsurgical pain with a dose- and time-dependent manner. Based on the fact that the effective doses of vitexin used in our study did not significantly reduce the spontaneous locomotor activity, we considered that the observed analgesic effects are not related to nonspecific behavioral suppression of vitexin. In previous studies^26^,^27^,^28^, it has been found that acute vitexin treatment increases the response latencies of mice in the hot-plate and tail-clip test, attenuates formalin-, capsaicin-, and complete Freund’s adjuvant (CFA)-induced defensive behaviors, ameliorates visceral pain in acetic acid- or PBQ-induced writhing test, and inhibits carrageenan- and capsaicin-induced mechanical and thermal hyperalgesia. Here we report an acute antinociceptive effect of vitexin against postoperative pain. Therefore, acute vitexin treatment possesses a wide spectrum analgesic ability in different pain conditions, implying the potential value of vitexin as an emerging analgesic for pain control.

It has been reported that repeated vitexin treatment attenuates CFA-induced chronic inflammatory pain without producing drug tolerance^28^. Therefore, next we examined the effect of repeated vitexin administration on the recovery of postoperative pain. We found that daily treatment with vitexin relieved postsurgical pain in a dose- and time-dependent manner. During the 3 days after surgery, the paw withdrawal threshold of mice that received no treatment maintained at a very low level (less than 0.6 g) and thereafter gradually returned to baseline level, which indicated that mice experienced severe postsurgical pain during this period. However, daily treatment with 10 mg/kg vitexin significantly increased the paw withdrawal threshold to a significantly higher level (close to 1 g) between 1 to 3 days after surgery, showing clear anti-hyperalgesic effects. This finding has significant clinical implications in that relieving severe postsurgical pain during the most painful days will reduce pain-induced adverse effects such as anxiety and increased risk of cardiovascular disorder, and facilitate patients’ recovery. It was also found that daily treatment with 10 mg/kg vitexin had no accumulative effect, consistent with the finding that the effect of acute 10 mg/kg vitexin treatment dissipated 4 h after administration. In a pharmacokinetic study, it was found that the elimination half-life of vitexin is relatively short (about 30 min) after intravenous administration in rats^32^, which seems to be consistent with the current study. Considering that postoperative pain usually has a well-defined pain period, these findings suggest that repeated vitexin treatment may be a useful strategy for the management of postoperative pain. In an effort to examine the potential preventive effect of vitexin treatment on postoperative pain, we found no significant effect in this regard even after daily treatment with vitexin for 6 days before the surgery. Thus, vitexin may have little value as a prophylactic agent for surgical pain.

So far, the information regarding the pharmacological mechanisms underlying the antinociceptive action of vitexin is very limited. In one study, it was reported that opioid mechanism is involved in vitexin-induced antinociception in assays of acute nociception (i.e., hot plate test and tail clip test) in healthy mice^27^. Here we examined this potential mechanism in postoperative pain. We found that a dose of 3 mg/kg opioid receptor antagonist naltrexone, which sufficiently occupies the majority of opioid receptors in mice, nearly completely blocked the anti-hyperalgesic effect of vitexin. This result strongly suggests that opioid receptors play a pivotal role in vitexin-induced analgesic effect. The descending monoamine pathway, especially the serotonin (5-hydroxytryptamine, 5-HT) system, plays an important role in endogenous pain modulation^33^. Currently, there are seven families of 5-HT receptors (5-HT_1–7_) that have been identified^34^ and at least five subtype receptors (5-HT_1/2/3/4/7_) are thought to be involved in the pain control^35^,^36^. In the dorsal horn of spinal cord, 5-HT_1A_ receptors are found at high concentrations, which are thought to exhibit inhibitory influence on neuronal excitability^33^. It seems contradictory that activation of 5-HT_1A_ receptors exerts both antinociceptive and pro-nociceptive effects in the spinal cord^37^,^38^. Nevertheless, in a postoperative pain model a high-efficacy 5-HT_1A_ receptor agonist F13640 was found to produce intra- and postoperative analgesic effect^39^. Recently, vitexin was found to have anti-depressant-like effect which may be related to the activation of 5-HT_1A_ receptors^21^. Thus, we examined the involvement of 5-HT_1A_ receptors in the vitexin-induced anti-hyperalgesic effects on postoperative pain. In this study, we found that pretreatment with WAY100635 at a dose sufficient to block 5-HT_1A_ receptors did not alter the antinociceptive effect of vitexin, suggesting that 5-HT_1A_ receptors are not involved in vitexin-induced analgesia. Accumulative evidence reveals that GABAergic neurons and GABA_A_ receptors play an essential role in pain perception and GABA_A_ receptors can serve as targets for developing novel analgesics in pathological pain^40^,^41^. More recently, GABAergic systems have also been found to regulate the incisional pain and GABA_A_ receptors are promising targets for postoperative pain^42^. Therefore, we next examined the possibility that GABA_A_ receptors are involved in vitexin-induced anti-hyperalgesic effect. We found that bicuculline, a selective GABA_A_ receptor antagonist, completely blocked a dose of 10 mg/kg vitexin-induced analgesia, suggesting that activation of GABA_A_ receptors also mediates vitexin-induced antinociception on postsurgical pain. Together, we conclude that both opioid receptors and GABA_A_ receptors are involved in vitexin-induced analgesia against incisional pain.

Acetaminophen is commonly used to treat postoperative pain and is a viable alternative to NSAIDs due to its lower incidence of adverse effects^43^. The strategy of combination of acetaminophen and other traditional analgesics is also frequently applied for pain management to achieve better therapeutic effects and/or increased safety profiles. For example, acetaminophen is found to confer additive analgesic effects in postoperative pain when it is used in combination with other analgesics such as morphine and NSAIDs^43^,^44^. In the present study, we also examined whether the combination of vitexin and acetaminophen could produce synergic or additive analgesic effects against postsurgical pain. Interestingly, the combination of vitexin and acetaminophen showed clear infra-additive or antagonistic interaction. Recent evidence indicated that the antinociceptive action of acetaminophen is antagonized by flumazenil, a benzodiazepine sensitive GABA_A_ receptor antagonist, which suggests GABA_A_ receptors play an important role in acetaminophen-induced analgesia^45^. Our study also reveals the involvement of GABA_A_ receptors in vitexin-induced analgesia. So, we postulated that acetaminophen may be a competitive antagonist for vitexin, since the two drugs both exert their effects through GABA_A_ receptors. This may tentatively explain the antagonistic interaction between them observed in our study. Further studies are required to confirm this possibility. Although the exact mechanism why an antagonistic interaction exists between vitexin and acetaminophen is unknown, these data do suggest that the combination of vitexin and acetaminophen for treating postoperative pain may not be a useful strategy.

In conclusion, the present study demonstrates that both acute and repeated vitexin treatment exert behaviorally-specific antinociception on postoperative pain which is primarily mediated through opioid receptors and GABA_A_ receptors. In addition, the combination of vitexin and acetaminophen exhibits an infra-additive effect, a finding that argues against the utility of such a combination therapy strategy. Combined, although repeated vitexin treatment has no preventive effect on postsurgical pain, our results strongly suggest that vitexin could be useful for the control of postoperative pain and deserves further investigation.

## Methods

### Animals

Adult male ICR mice weighing 18–22 g (Laboratory Animal Center, Nantong University, Nantong, China) were acclimatized in groups (4–5 per cage) for at least 3 days under constant conditions of temperature (22 ± 1 °C) and humidity (50–70%) with a 12-hour light–dark cycle (lights on 7:00 AM). Animals had free access to food and water except during experimental sessions. All experimental procedures were approved by Nantong University Committee on Animal Care and Use and animals were maintained in accordance with the *Guide for the care and use of laboratory Animals* (8^th^ edition, Institute of Laboratory Animal Resources on Life Sciences, National Research Council, National Academy of Sciences, Washington, DC). All animal handlings complied with the recommendations of the International Association for the Study of Pain and all efforts were made to minimize the number of animals used and their suffering.

### Drugs

Vitexin and acetaminophen were purchased from Aladdin Reagents (Shanghai, China) and were suspended in 0.5% carboxymethylcellulose sodium. Naltrexone hydrochloride, WAY100635 (N-[2-[4-(2-methoxyphenyl)-1-piperazinyl]ethyl]-N-(2-pyridyl) cyclohexanecarboxamide), and bicuculline were purchased from Selleck Chemicals (Houston, TX, USA) and were dissolved in 0.9% physiologic saline. All drugs were administered intraperitoneally (i.p.) in a volume of 10 ml/kg of body weight.

### Incisional surgery

The mouse model of hindpaw incisional surgery was performed as previously described^30^,^31^. Briefly, mice were anesthetized with 2% to 3% isoflurane delivered via a nose cone. After sterile preparation, a 5 mm longitudinal incision was made with a number 11 blade on the plantar surface of the right hind paw, starting 2 mm from the proximal edge of the heel and extending toward the toes. The plantaris muscle was elevated and incised longitudinally. After controlling bleeding through gentle pressure, a single 6–0 nylon suture was placed through the midpoint of the incision. The animals were allowed to recover in their home cages.

### Von Frey filament test

As described before^31^, the mechanical hyperalgesia was measured by assessing paw withdrawal thresholds (PWT) to mechanical stimuli using von Frey filaments (Stoelting, USA). Seven calibrated von Frey filaments with approximately equal logarithmic incremental bending forces (i.e., 0.07, 0.16, 0.4, 0.6, 1.0, 1.4, 2.0 g bending force) were used. Mice were acclimated on an elevated wire mesh floor covered with a clear Plexiglas chamber for at least 20 min, and then the fibers of sequentially increasing stiffness with initial bending force of 0.07 g were applied to the plantar surface of the hindpaw adjacent to the incision for 5 sec with enough force to slightly bend the fiber. Each filament was tested three times per paw, and the PWT was defined as the minimal force that caused at least two withdrawals observed out of three consecutive trials. If a mice did not produce withdrawal response to the filament with maximal force 2.0 g, the PWT was record as 2.0 g.

### Locomotor activity

The locomotor activity of mice was measured by a commercially available apparatus (YLS-1B, Shandong Academy of Medical Sciences, China), which consists of a controller unit and four circular black acrylic locomotion units (30 cm in diameter and 30 cm in height). The spontaneous locomotor activity of the mice was recorded through the photocell sensor located in the center of the cover. Locomotor activity was measured by the beam breaks (counts) during a 60-min test period.

### Experimental design

For the measurement of mechanical hyperalgesia, the PWT was measured 2 h after the surgery and daily thereafter for 6 days. Daily baseline measures prior to surgery were performed for 3 days to facilitate mice to habituate to the procedure and experimenters. For acute effect of vitexin, four doses of vitexin (1–10 mg/kg) were used and the drug was administered intraperitoneally (i.p.) 1 day post-surgery. After treatment, the PWT was measured every 30 min for 4 hours. For repeated treatment study, three doses of vitexin (3–10 mg/kg) were studied. The drug was administered to mice 1 day after surgery and once daily for 6 days. The PWT was measured 90 min after the administration of the drug [results from acute treatment tests indicated that 90 min is the time to achieve maximal antinociceptive effect for vitexin (Fig. 2)]. In order to examine potential accumulating effects of vitexin, the PWT was also measured prior to drug administration between 2 to 4 days after surgery during the period that mice received daily 10 mg/kg vitexin treatment. For prevention study, mice received once daily vitexin treatment prior to surgery for 6 days, and then the PWT of mice was measured 2 h post-surgery and thereafter once daily for 6 days. For antagonist studies, one day after surgery, different antagonists were administered 10 min prior to 10 mg/kg vitexin administration. The von Frey test was performed every 30 min for 4 hours after treatment of vitexin. Blind design was strictly followed for all the studies such that different experimenters conducted drug treatments and behavioral measures.

### Data analyses

All data were presented as mean ± standard error of mean (SEM) and were analyzed using the GraphPad Prism 5.01 software (San Diego, CA, USA). For anti-hyperalgesia studies, the PWT (gram) was plotted as a function of time (min or days). Statistical differences between groups in the von Frey test were analyzed by a two-way analysis of variance (ANOVA) method with repeated measures using time as within group factor and different doses of vitexin or different antagonists as between group factor, which was followed by Bonferroni post hoc analysis. For locomotor activity test, group differences in total locomotion counts within 60 min were evaluated using one way ANOVA followed by Student–Newman–Keuls post hoc analysis. For all analyses, statistical significance level was set at P < 0.05. For the drug combination test, the anti-hyperalgesic effect of drugs was quantified as the percentage of the maximum possible effect (MPE) according to the following equation: MPE % = [(post-drug PWT—pre-drug PWT)/(pre-surgery baseline PWT—pre-drug PWT)] × 100. The dose-response curves for vitexin and acetaminophen alone were constructed by plotting the maximal effects of individual doses and the individual ED_50_ (±SEM) values were respectively determined using the GraphPad Prism 5.01 software (San Diego, CA, USA). In order to examine the interaction between vitexin and acetaminophen, an isobolographical analysis method was used as described in our previous study^46^. Briefly, vitexin and acetaminophen were combined in a fixed ratio proportions of the equieffective ED_50_ dose for vitexin and acetaminophen (1:1) and studied in separate groups of animals. Then the dose-response curves for vitexin and acetaminophen in the drug mixture were constructed by plotting the maximal effects of individual doses and the individual ED_50_ values of the two drugs in the mixture were calculated. Finally, an isobologram was constructed to visually reveal the drug interactions as supra-additive, additive, or infra-additive. The isobologram was constructed by connecting the ED_50_ values of acetaminophen plotted on the abscissa with the ED_50_ values of vitexin plotted on ordinate to obtain the line of additivity. The 95% confidence intervals (CIs) of ED_50_ for vitexin and acetaminophen alone were also connected to obtain the global 95% confidence boundaries, indicating the limits of the additive line. If the effects of the two drugs are additive, the ED_50_ values (±SEM) for the drug combination should fall within the limits of the additive line. If the ED_50_ values (±SEM) fall below the limits of the line of additivity, the effects of the two drugs are considered to be supra-additive or synergistic. If the ED_50_ values (±SEM) fall above the limits of the additive line, then the effects of the two drugs are considered to be infra-additive.

## Additional Information

**How to cite this article**: Zhu, Q. *et al*. Antinociceptive effects of vitexin in a mouse model of postoperative pain. *Sci. Rep.* **6**, 19266; doi: 10.1038/srep19266 (2016).
